# Micro-optical coherence tomography of the mammalian cochlea

**DOI:** 10.1038/srep33288

**Published:** 2016-09-16

**Authors:** Janani S. Iyer, Shelley A. Batts, Kengyeh K. Chu, Mehmet I. Sahin, Hui Min Leung, Guillermo J. Tearney, Konstantina M. Stankovic

**Affiliations:** 1 Eaton-Peabody Laboratories and Department of Otolaryngology, Massachusetts Eye and Ear Infirmary, 243 Charles St, Boston, MA, USA; 2 Department of Otolaryngology, Harvard Medical School, 25 Shattuck St, Boston, MA, USA; 3 Program in Speech and Hearing Bioscience and Technology, Harvard University Graduate School of Arts and Sciences, 1350 Massachusetts Ave, Cambridge, MA, USA; 4 Wellman Center for Photomedicine, Massachusetts General Hospital, 50 Blossom St, Boston, MA, USA; 5 Department of Pathology, Massachusetts General Hospital, 55 Fruit St, Boston, MA, USA

## Abstract

The mammalian cochlea has historically resisted attempts at high-resolution, non-invasive imaging due to its small size, complex three-dimensional structure, and embedded location within the temporal bone. As a result, little is known about the relationship between an individual’s cochlear pathology and hearing function, and otologists must rely on physiological testing and imaging methods that offer limited resolution to obtain information about the inner ear prior to performing surgery. Micro-optical coherence tomography (μOCT) is a non-invasive, low-coherence interferometric imaging technique capable of resolving cellular-level anatomic structures. To determine whether μOCT is capable of resolving mammalian intracochlear anatomy, fixed guinea pig inner ears were imaged as whole temporal bones with cochlea *in situ*. Anatomical structures such as the tunnel of Corti, space of Nuel, modiolus, scalae, and cell groupings were visualized, in addition to individual cell types such as neuronal fibers, hair cells, and supporting cells. Visualization of these structures, via volumetrically-reconstructed image stacks and endoscopic perspective videos, represents an improvement over previous efforts using conventional OCT. These are the first μOCT images of mammalian cochlear anatomy, and they demonstrate μOCT’s potential utility as an imaging tool in otology research.

Few treatments for human hearing loss exist, largely because the relationship between an individual patient’s cochlear pathology and their degree of hearing loss is poorly understood. A large obstacle to achieving this understanding is the inability to perform noninvasive imaging on patients’ inner ears at a resolution sufficient to assess potential physiological contributions to hearing impairment. Hearing loss can result from physiological damage to the sensory hair cells and spiral ganglion, malformation of or damage to areas necessary for sound conduction through bone, or a mixture of these pathologies^1^. However, conventional clinical imaging methods, such as magnetic resonance imaging (MRI) and computed tomography (CT), are limited in spatial resolution to approximately 1 mm and 0.5–1 mm, respectively^2^,^3^. Consequently, these modalities can only detect gross abnormalities, such as profound malformations in the bony anatomy of the cochlea. MRI and CT are largely insensitive to intracochlear defects that fall beneath this detection range, such as missing and damaged cells within the cochlea’s sensory epithelium, the organ of Corti^4^.

The organ of Corti is a heterogeneous matrix of sensory and non-sensory epithelial cells that contribute to both the perception and fine-tuning of frequencies within the range of mammalian hearing^5^. Supporting cells, such as pillar, Deiters, and Hensen’s cells, provide structural and molecular support to the sensory hair cells. Hair cells are organized as a single row of inner hair cells (IHC), which receive 90% afferent innervation, and three rows of outer hair cells (OHC), which receive 90% efferent innervation. The inner hair cells transduce sound via mechanical shearing forces imparted by the basilar membrane’s vibration and the cells’ protruding actin stereocilia. Stereocilia deflection prompts neurotransmitter release into the post-synaptic space near spiral ganglion neurites, generating electrical signals in response to frequency-specific stimulation that is sent to brainstem and cortical processing regions. The cochlea contains other soft tissue microstructures that are critical for hearing: Reissner’s membrane, which serves as a diffusion barrier separating the contents of two of the cochlea’s fluid-filled cavities; the tectorial membrane, which contacts hair cells’ stereocilia during sound transduction; the stria vascularis in the spiral ligament, which maintains ionic gradients of endocochlear fluids and provides a blood barrier; and the neurites of the spiral ganglion, which form one branch of the auditory nerve, and extend both radially and diagonally along the sensory epithelium^6^. These structures fall beneath the detection limits of both MRI and CT.

Optical coherence tomography (OCT) is a non-contact, cross-sectional imaging technique that applies low coherence interferometry to image opaque subsurface structures with a resolution typically from 10–15 μm in axial and 30–40 μm in transverse planes^7^,^8^. During OCT imaging, infrared laser light is backscattered by microstructural features within a structure or organ of interest. The dimensions of these features can be determined by applying low coherence interferometry, which enables the backscattered sample light to be resolved in depth. OCT is characterized by high detection sensitivity, as small as 10^−10 ^of the incident optical power^8^, and a penetration depth of 1–3 mm, depending on tissue type^7^. OCT is routinely used in clinical ophthalmology to image the retina and cornea^9^,^10^ and in dermatology^11^,^12^, and has previously been used for cellular and submicrometer imaging^13^,^14^,^15^. Intracochlear morphology and mechanics have also been observed with OCT in rodent models, with axial and lateral resolution ranging from 10–20 μm^16^,^17^,^18^,^19^,^20^, *ex vivo*^16^,^17^,^19^ and *in vivo*^13^,^18^,^20^. OCT has enabled the identification of larger structures including Reissner’s membrane^16^,^17^,^18^,^19^,^20^, the basilar^16^,^17^,^18^,^19^,^20^ and tectorial membranes^18^,^19^,^20^, the spiral ligament^17^, the three scalae of the cochlea^16^,^18^,^20^, and the region of the sensory epithelium^18^,^19^,^20^, in addition to spaces between various structures such as the demarcation between the osseous and membranous labyrinths^17^, the tunnel of Corti^19^, and spaces between regions of inner and outer hair cells^19^, *ex vivo*^16^,^17^,^19^ and *in vivo*^18^,^20^. Cochlear mechanics and measurements of the motion and displacement of intracochlear structures in response to frequency-specific auditory stimulation have also been studied with OCT *ex vivo*^21^,^22^ and *in vivo*^20^. However, similar to traditional imaging methodology, these studies have been limited by OCT’s resolution threshold, and have not been capable of resolving smaller anatomical features including the major therapeutic targets in hearing loss such as inner and outer hair cells, supporting cells, and nerve fibers.

To improve the spatial resolution of conventional OCT, we introduced a successor called micro-optical coherence tomography (μOCT) and demonstrated its ability to resolve individual endothelial cells, leukocytes, lymphocytes, and monocytes in human cadaver coronary arteries, at a resolution of 2 μm × 2 μm × 1 μm (x, y, z)^23^. μOCT has more recently been utilized to detect cholesterol crystals within macrophages in atherosclerosis^24^, to visualize functional anatomy, including individual beating cilia involved in mucociliary clearance and transport in airway epithelium^25^,^26^, and to resolve cellular details in zebrafish larvae *in vivo*^27^. μOCT technology may also be suitable to resolve cochlear microanatomy at a cellular level. Thus, this study’s objective was to determine whether μOCT was capable of resolving major and micro-anatomical structures within the mammalian inner ear, and to generate the first μOCT images of fixed guinea pig intracochlear anatomy *in situ*.

## Results

**Figure 1:**
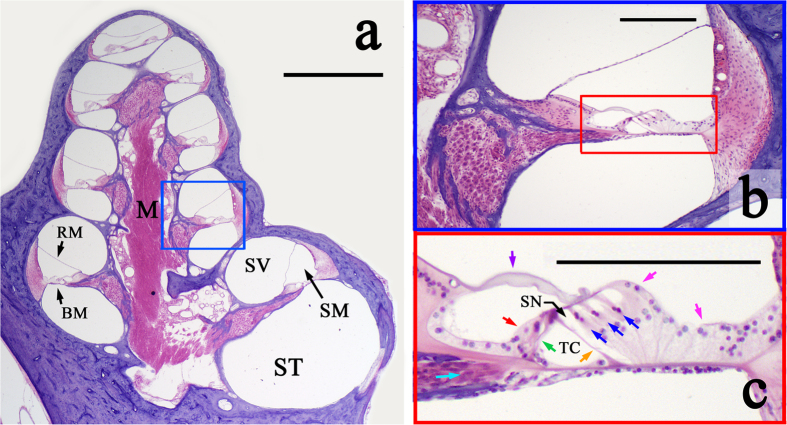
Micrographs of a sectioned guinea pig cochlea. **(a)** Cross-section of a guinea pig cochlea, stained with H&E, and cut along its longitudinal axis. M: modiolus. BM: basilar membrane; RM: Reissner’s membrane, ST: scala tympani; SM: scala media; SV: scala vestibuli. Magnification = 2×; Scale = 1 mm. **(b)** A single half-turn of the guinea pig cochlea, representing the region boxed in blue in (**a**). Magnification = 10×; Scale = 200 μm. **(c)** Zoomed-in view of the organ of Corti (boxed in red in (**b**)). Colored arrows point to specific cell types: inner (red) and outer (blue) hair cells, inner (green) and outer (orange) pillar cells, and neuronal fibers (turquoise), which travel through the tunnel of Corti (TC) and space of Nuel (SN). Pink arrows: supporting cells; purple arrow: tectorial membrane. Magnification = 10×; Scale = 200 μm.

Figure 1a–c depicts cross-sections of a guinea pig cochlea cut along its longitudinal axis, and stained with hematoxylin and eosin (H&E) to highlight cellular structures. Figure 1a shows eight cochlear half-turn cross-sections, corresponding to the four cochlear turns, spiraling around the bony, neuron-filled core, the modiolus (M). The basilar membrane (BM) and Reissner’s membrane (RM) delineate the cochlea’s three fluid-filled chambers: the scala tympani (ST), scala media (SM) and scala vestibuli (SV). The bony otic capsule surrounding the cochlear tissue is stained purple. Figure 1b zooms in on the single half-turn (boxed in blue in Fig. 1), and Fig. 1c zooms in on the region boxed in red in Fig. 1b, depicting the sensory cells (inner and outer hair cells) and non-sensory cells (including inner and outer pillar cells) of the organ of Corti, as well as other supporting cells and the tectorial membrane.

**Figure 2:**
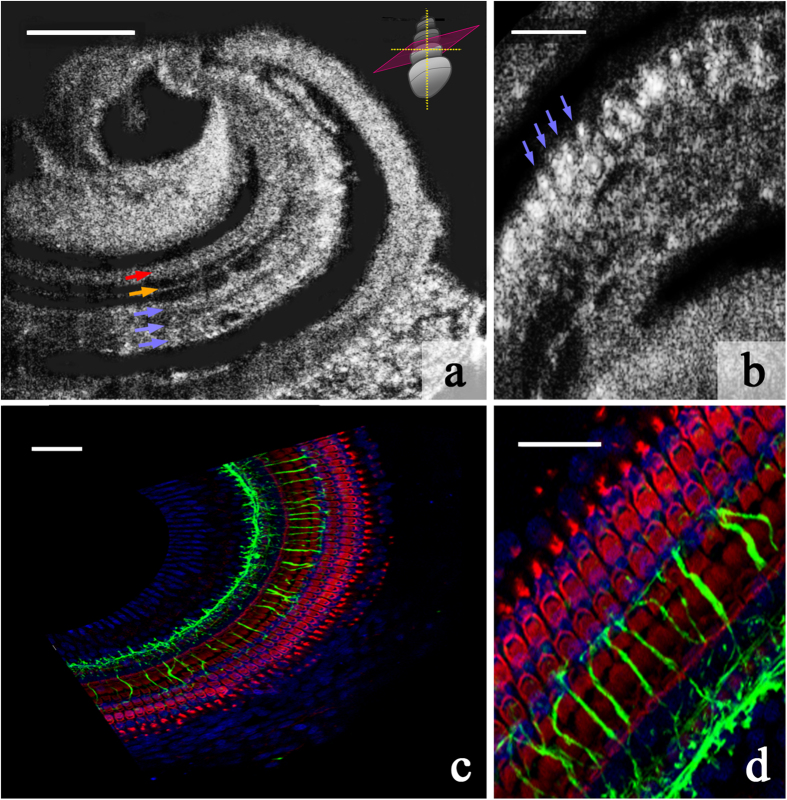
μOCT images and immunohistochemically-stained regions of sensory and supporting cell rows within the guinea pig organ of Corti. (**a**) Single 2D image from a μOCT imaging stack, depicting the regions of the inner pillar cells and inner hair cells (red arrow), outer pillar cells (orange arrow), and 3 rows of outer hair cells (blue arrows). The schematic in the top right-hand corner shows the orientation of the plane (pink) along which the image was sectioned relative to the orientation of the cochlea. Scale = 100 μm. (**b**) Single 2D image depicting individual outer hair cells (examples indicated with blue arrows). Scale = 50 μm. (**c**) Immunohistochemically-stained guinea pig organ of Corti whole mount, corresponding to the orientation presented in (**a**). Cytoskeletal actin within hair cells and supporting cells is labeled with rhodamine phallodin (red), neuronal processes are labeled with neurofilament-H (green), and cell nuclei are labeled with Hoechst stain (blue). Scale = 50 μm. (**d**) Zoomed-in view of immunohistochemically-stained guinea pig organ of Corti depicting the orientation presented in (**b**). Color convention as in (**c**). Scale = 25 μm.

Excised guinea pig temporal bones were imaged with μOCT via a 0.5–1 mm diameter cochleostomy in the otic capsule, corresponding to either a) the region of the second of the four cochlear turns, exposing the area from the top of the third turn to the top of the second turn, laterally, or b) the apex. μOCT permitted imaging in 1 mm × 1 mm and 500 μm × 500 μm fields of view. Raw images of the cochlea’s apical turn are shown in Fig. 2. Figure 2a reveals the region of inner hair cells and inner pillar cells, a row of outer pillar cells (OPCs), and three rows of OHCs. The dark space between the OPCs and OHCs is the space of Nuel; the dark space between the OPCs and the inner pillar cells is the tunnel of Corti. Figure 2b shows a single plane image of the region where outer hair cells reside – individual outer hair cells are identifiable. For reference and orientation, Fig. 2c,d show immunohistochemically-labeled cells and neuronal processes in the guinea pig organ of Corti.

**Figure 3:**
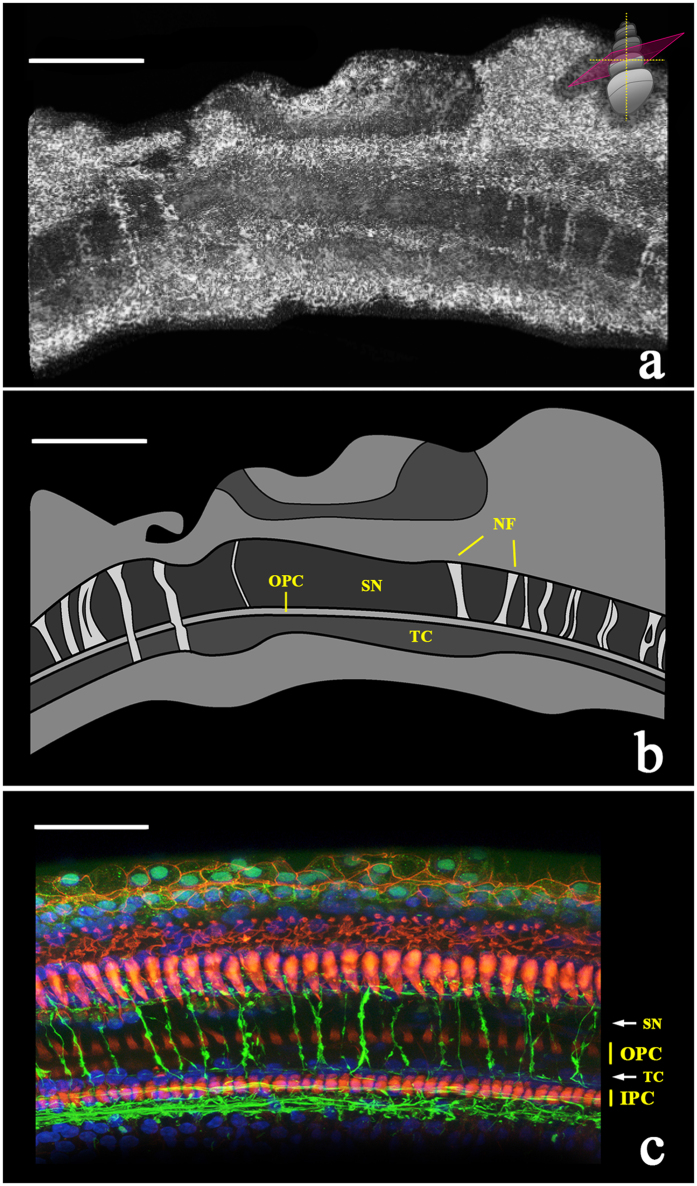
μOCT image of nerve fiber bundles traversing the tunnel of Corti and space of Nuel to innervate outer hair cells (500 μm × 500 μm). (**a**) Volumetric reconstruction of maximum-projected μOCT image stack, depicting bundles of nerve fibers traversing the organ of Corti towards the outer hair cell region. The schematic in the top right-hand corner shows the orientation of the virtual sectioning plane. Scale = 150 μm. (**b**) Schematic representation of the microanatomy in the top panel, with bundles of nerve fibers (NF) crossing the tunnel of Corti (TC) and/or the space of Nuel (SN). OPC = outer pillar cells. Scale = 150 μm. (**c**) For reference, a confocal laser scanning microscopy image of the guinea pig organ of Corti. Rhodamine phalloidin (red) marks outer and inner pillar cells (OPC and IPC, respectively), Hoechst stain (blue) marks cell nuclei, and neurofilament-H (green) marks neuronal fibers. Scale = 50 μm.

After performing a volumetric reconstruction of the raw 2D scans, neuronal fiber bundles became visible at several levels within the tunnel of Corti and space of Nuel along the length of the imaged tissue (Fig. 3). Due to their location and radial trajectory across the tunnel of Corti and space of Nuel (the endolymph-filled epithelial lumens situated between the inner and outer pillar cells and the outer pillar and hair cells, respectively), these nerve fiber bundles are hypothesized to be synaptically connected to outer hair cells.

**Figure 4:**
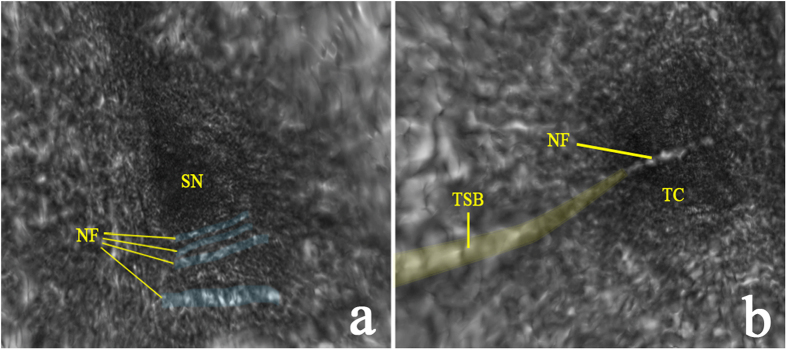
3D volumetric reconstruction revealing bundles of nerve fibers traveling through the tunnel of Corti and space of Nuel. (**a**) A labeled, colorized still from a 3D volumetric reconstruction of a μOCT image stack (Visualization 1a), “flying through” the space of Nuel (SN), showing bundles of nerve fibers (NF, blue) crossing the basal region of the SN. (**b**) A labeled, colorized still from a 3D volumetric reconstruction of a μOCT image stack (Visualization 1b), “flying through” the tunnel of Corti (TC), showing a single bundle of medial efferent nerve fibers crossing the central region of the TC, and the tunnel spiral bundle (TSB, yellow) running along the tunnel’s medial wall. Please refer to the Supplemental Materials to view Visualizations 1a and b. 500 μm × 500 μm field of view.

**Figure 5:**
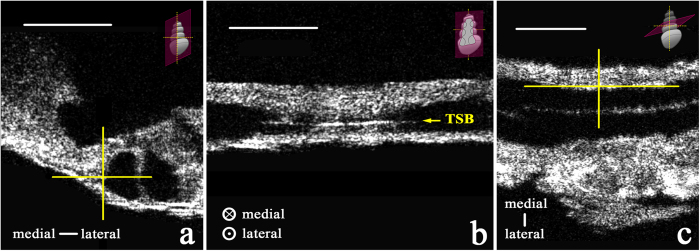
Two-dimensional μOCT images of the tunnel spiral bundle (TSB) (500 μm × 500 μm) within the guinea pig organ of Corti, in three perspectives. Yellow cross hairs in (**a**) (cross-section of the organ of Corti and two fluid lumens, separated by a pillar cell) and (**c**) (looking down from above the organ of Corti) indicate the TSB’s (medial section, shown in (**b**)) specific position along the medial wall of the tunnel of Corti. The schematics in the top right-hand corner of each panel show the orientation of the 2D plane depicted, respectively. Scale = 100 μm.

Visualizations 1a and 1b allow the viewer to virtually “fly through” the space of Nuel and tunnel of Corti, respectively, in volumetric reconstructions of two μOCT imaging stacks of the guinea pig organ of Corti. The orientation visualized here is the same as those depicted in Fig. 1a–c. In Visualization 1a, bundles of neuronal fibers are observed traversing the basal region of the space of Nuel (labeled still image shown in Fig. 4a). In Visualization 1b, a single bundle of neuronal fibers is observed crossing the central region of the tunnel of Corti (labeled still image shown in Fig. 4b). We hypothesize that this is a fascicle of medial olivocochlear efferent nerve fibers, based on its radial trajectory and location within the tunnel^28^. A different nerve fiber bundle is observed traveling longitudinally along the medial wall of the tunnel of Corti for the length of the reconstructed tissue section. The longitudinal trajectory and specific location of this bundle are characteristic of the tunnel spiral bundle (TSB), a mass of primarily lateral olivocochlear nerve fibers. The TSB is also visualized in cross-section in Fig. 5a–c, which display its precise location in three 2D orientations. Our interpretation regarding the tunnel spiral bundle and tunnel-crossing bundle of medial efferent fibers are consistent with previously reported arborization patterns of efferent fibers in the cat cochlea^29^,^30^.

**Figure 6:**
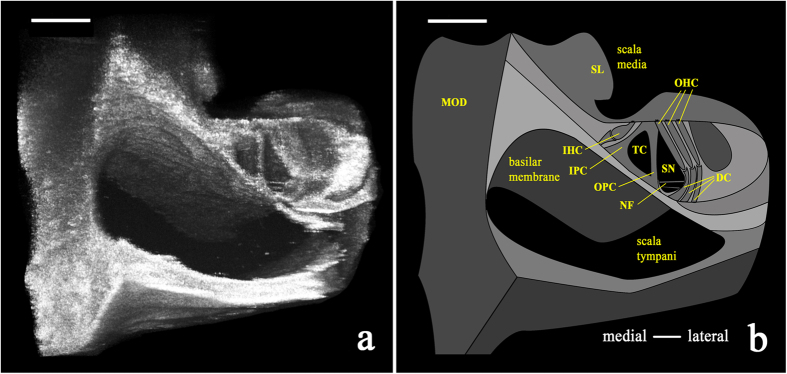
Volumetrically reconstructed μOCT image (500 μm × 500 μm) and schematic of the guinea pig organ of Corti *in situ*. (**a)** Volumetric reconstruction of μOCT-visualized sensory and non-sensory cells of the organ of Corti from the 2^nd^–3^rd^ turn of the cochlea. (**b)** Schematic labeling structures visualized in the left panel, such as outer hair cells (OHC), bundles of nerve fibers (NF), and inner and outer pillar cells (IPC and OPC, respectively). MOD = modiolus; SL = spiral limbus; IHC = inner hair cell; TC = tunnel of Corti; SN = space of Nuel. Both scales = 100 μm.

A volumetric reconstruction of the imaged section of the guinea pig organ of Corti viewed in cross-section revealed discernable individual cell types *in situ* (Fig. 6). In this image, the scalae tympani and media are clearly visualized, separated by the basilar membrane and the organ of Corti atop it. Other identifiable structures include the bony modiolus (MOD) and the spiral limbus (SL; medial and medio-apical to the basilar membrane, respectively), inner and outer pillar cells (IPC and OPC, respectively), outer hair cells (OHC), the tunnel of Corti (TC), and space of Nuel (SN; inferior and medial to the outer hair cells). The inner hair cells are medial to the tunnel of Corti; their embedded location did not permit visualization here. Bundles of nerve fibers (NF) were observed traveling from within the spiral lamina across the space of Nuel (SN) to the region of the outer hair cells.

## Discussion

In presenting the first, to our knowledge, μOCT images of the mammalian cochlea, this report provides evidence of μOCT’s utility as a high-resolution intracochlear imaging tool. Our μOCT imaging system resolved cellular anatomy in the guinea pig organ of Corti, including nerve fiber bundles, which have eluded conventional clinical imaging methods thus far. Importantly, results of the present study also reveal that high resolution is not the sole criterion for achieving informative images; indeed, our data suggest that μOCT resolves some of the organ of Corti’s anatomical and cellular features, such as lumens and bundles of neurites, more readily than other structures that are more deeply embedded in the sensory epithelium, such as inner hair cells. Thus, it is evident that factors such as sample contrast and speckle noise, in addition to resolution, are important consideration for future improvements on this technology^31^.

Hearing loss is the most common sensory deficit in the world^32^ and the most common disability in the United States^33^. Because mammalian cochlear hair cells and neurons do not spontaneously regenerate, hearing loss is permanent and irreversible in the vast majority of cases. A significant barrier to developing otologic therapies is a limited understanding of how cochlear pathology relates to the degree and type of hearing loss^34^. The cochlea remains a “black box” in living subjects, closed to direct or conventional imaging due to its embedded location, fragility, and complex structure. Our knowledge of cochlear physiology and morphology today thus comes primarily from post-mortem analyses of human temporal bones and experiments using animal models, which have revealed many physiological sources of hearing impairment: sensory hair cell loss or damage^35^,^36^; damage to stereocilia due to noise over-exposure^37^; malformations of the tectorial membrane^38^; loss of auditory nerve fibers and spiral ganglion neurons^39^; and atrophy of the stria vascularis^40^.

Numerous studies have noted the value of traditional OCT imaging for the cochlea^16^,^17^,^18^,^19^,^20^,^21^,^41^,^42^,^43^; however, the improvements that μOCT affords over OCT in resolution and depth-of-focus (DOF) make it better-suited for imaging the cochlea. The anatomical undulations in the cochlea require high DOF to simultaneously capture peaks and valleys; OCT’s shorter DOF may be responsible for limiting earlier studies to 5–10 μm resolution. We have previously reported concurrent gains in DOF and resolution in μOCT and established the utility of this enhanced performance in cardiovascular^23^ and airway imaging applications^44^. Compared to other conventional imaging, the advantages of μOCT include that it (1) can be conducted on the benchtop, (2) requires no contrast agent, (3) can image whole structures within its detection field near-instantaneously, and (4) employs primarily infrared and near-infrared light sources, theoretically safer than higher-energy (lower wavelength) laser exposure^45^. While significant development is required before this technology can be employed to assess cochlear pathology in living humans, the present study demonstrates μOCT’s potential to image this organ and motivates further miniaturization of μOCT technology. We have recently reported progress in the development of miniaturized μOCT instrumentation for *in vivo* applications^46^,^47^ and in human cochlear endoscopy via the external auditory canal^48^. However, the adaptation of our imaging configuration to the constraints of the small size and embedded location of the human cochlea remains a significant technical challenge.

Clinical realization of μOCT cochlear imaging faces significant surgical and engineering hurdles; however, applications for otolaryngology research in animals may be more immediately realized. In contemporary studies employing animal models of hearing impairment, the techniques most commonly used to detect and visualize changes in cochlear morphology include confocal and two-photon microscopy in tandem with histology and immunohistochemistry. These techniques are applied post-mortem, and both specimen preparation and imaging itself may be extremely time-consuming. An imaging technique such as μOCT, capable of resolving cochlear microanatomy in experimental animals as genetic models of human hearing loss, could potentially be adapted for *in vivo* longitudinal analysis of cochlear anatomy in healthy and pathological states. Potential *in vivo* applications in animals include monitoring physiological changes resulting from genetic mutations associated with progressive hearing loss, facilitating surgical guidance when advancing therapy-delivering probes, targeting distinct intracochlear spaces or frequency-specific locations through narrow cavities in the ear^49^, assessing and measuring vibration in the organ of Corti prior to and post exposure to noise or regenerative therapies^50^, and acquiring an improved understanding of the natural progression of age-related hearing loss. Future directions of this work include conducting *in vivo* μOCT experiments in guinea pigs, to determine optimal surgical approaches and the instrumentation’s performance in a living subject.

The present work is subject to several limitations, namely that the described experiments were conducted in an animal model and within healthy, normal cochleae; the specimens used were excised, partially dissected, and fixed for ease of manipulation; and a small number of cochleae (7) were examined. Nevertheless, these findings represent an important incremental advance regarding μOCT’s ability to perform cellular-level imaging of the mammalian cochlea, which we hope will accelerate the technology’s improvement and customization for broader applications.

## Conclusions

Innovation of imaging systems capable of resolving mammalian middle and inner ear anatomy is essential for understanding the link between otopathology and hearing function. μOCT, shown here to be capable of resolving microanatomy in the mammalian cochlea, affords the resolution and speed necessary to be considered a promising candidate for otologic imaging development; however, significant progress remains to be made. Future efforts should aim to improve μOCT’s resolution and penetration depth, determine whether similar imaging results can be reproduced in living animal subjects, and define the limits of the instrumentation and surgical approaches to permit optimal imaging access.

## Methods

### Animals

The guinea pig is a well-studied animal model that is commonly used in translational research studies on hearing and hearing loss because its frequency sensitivity and susceptibility to ototoxic medications are similar to that in humans^51^,^52^,^53^,^54^, and its entire cochlea is surgically accessible. Albino adult male guinea pigs of approximately 400 grams were used in this study (7 cochleae were imaged). The Institutional Animal Care and Use Committee (IACUC) of Massachusetts Eye and Ear Infirmary (MEEI) approved all experimental protocols for this study, and all procedures were carried out in accordance with approved institutional guidelines of the IACUC. Guinea pigs were maintained at MEEI’s animal care facility in Boston, MA.

### Instrumentation and image collection

**Figure 7:**
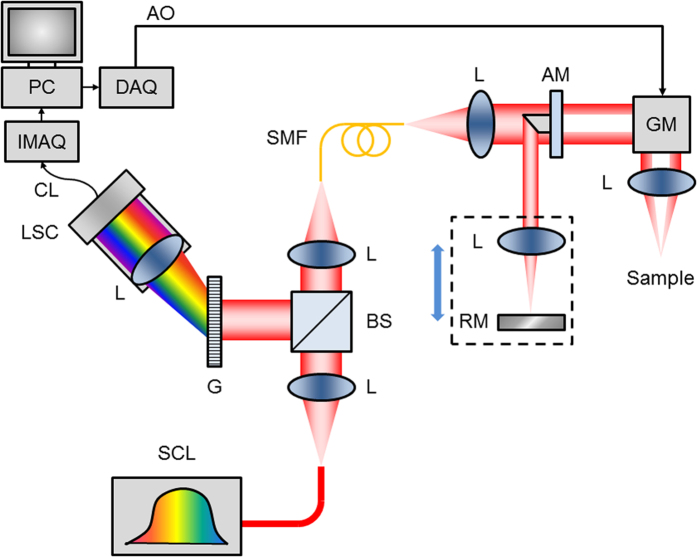
μOCT instrumentation. Schematic diagram of μOCT system. Supercontinuum laser (SCL) power is directed by collimating and focusing lenses (L) through a single mode fiber (SMF). Output light from the SMF is collimated and passed through an apodizing mirror (AM), resulting in a circular obscuration of the transmitted light, which is steered by a galvanometer mirror (GM) through an objective lens onto the sample. Light reflected by the AM is focused onto a reference mirror (RM), and the reference lens and mirror assembly can be translated in unison to adjust the reference path length. Back-scattered light from the sample is re-integrated at the SMF with light reflected by the RM. The return light is collimated and directed by the beam-splitter (BS) towards a diffraction grating (G). The spectrally dispersed light is then focused onto a line scan camera (LSC), which outputs raw spectrograms through a CameraLink (CL) interface to an image acquisition board (IMAQ) installed in a PC. The PC also controls scanning through a data acquisition card (DAQ), which produces an analog output (AO) voltage signal that controls the GM.

The system was a customized, spectral-domain OCT (SD-OCT) instrument with improvements to standard OCT that yield higher resolution in lateral and axial directions. The instrumentation layout has been previously described^23^ and is illustrated in Fig. 7.

In brief, a circular obscuration was created in the sample beam path to enhance axial depth-of-focus (DOF) and field-of-view, maintaining an extended DOF of approximately 300 μm with a numerical aperture of 0.12. The resulting lateral resolution was 2 μm^23^. The high axial resolution of 1 μm was derived from a high bandwidth custom OCT spectrometer, spanning 650–950 nm combined with an ultra-broadband supercontinuum laser source (NKT Photonics, Birkerod, Denmark).

The optical power at the sample was less than 15 mW. Transverse (x, y) scanning across the sample was performed using software-controlled galvanometer scanning motors (Thorlabs, Newton, NJ); the same software was used to simultaneously acquire spectral data through the spectrometer camera. Images comprising 512 A-lines were acquired at 40 frames per second (fps), with each frame spanning either 500 μm or 1 mm in lateral space. Three-dimensional (3D) volumes were acquired by scanning over a square region spanning either 500 × 500 μm or 1 × 1 mm. A large working distance (25 mm), defined as the distance between the objective lens and the focal plane, was chosen to allow flexible positioning of specimens on the sample stage. Cross-sectional and 3D images are displayed using logarithmic, logarithmic inverse, or linear grayscale lookup tables, which depict liquid-filled regions (e.g. the scalae) as dark, and highly-scattering regions (e.g. bone and tissue) as light.

### Specimen processing

Guinea pig temporal bones were extracted following euthanasia (intraperitoneal injection of Fatal-Plus Solution [0.1 mL/kg; Vortech Pharmaceuticals, Dearborn, MI]) and decapitation. Temporal bones were dissected in cold 4% paraformaldehyde (PFA) (diluted in Phosphate Buffered Saline [PBS]) to expose the cochlea’s interior to fixative by opening the apical otic capsule and puncturing the round and oval window membranes with forceps. Approximately 1 ml of cold 4% PFA was slowly infused into the apical hole until it flowed out of the oval and round windows. The temporal bones were immersed in cold 4% PFA in PBS for 4–8 hours on a shaker. Seven specimens were further dissected in PBS to remove some of the otic capsule, exposing a section or the entire cochlea. Specimens were stored at 4 °C in PBS prior to μOCT imaging. Two additional specimens were placed in 0.12M EDTA for two weeks to decalcify the otic capsule for whole mount preparation and immunostaining (see section ‘Immunohistochemistry’ below).

### μOCT imaging and image analysis

During μOCT imaging, specimens were removed from the PBS and positioned beneath the μOCT laser aperture in a dry plastic culture dish. The imaging apparatus was adjusted in X, Y, and Z directions to achieve optimal focus.

Raw data were converted into tagged image file format (.tif) stacks using standard SD-OCT processing routines^55^ and then imported into OsiriX (Pixmeo SARL, Bernex, Switzerland), a software program routinely used to analyze clinical CT and MRI images. OsiriX was used to reconstruct (multiplanar reconstruction and volumetric rendering) the μOCT images in 3D, generate maximum intensity projections, rotate, enlarge, and crop the images, set image opacity, and generate scale bar measurements. Images were labeled, structures were colorized with a partially transparent brush (for ease of visualization), and scale bars were traced in Photoshop (Adobe, Inc., San Jose, CA). 3D volumetric μOCT images were constructed using an endoscopy perspective in OsiriX to create “fly-through” videos (15 fps) revealing intracochlear structures.

### Haematoxylin/eosin (H&E) histology

Animal euthanasia and specimen extraction and fixation followed the protocol described above. For H&E staining, guinea pig cochleae were perfused with 10% formalin and decalcified in 0.27M EDTA for 25 days. Specimens were then dehydrated in ethanol and embedded in celloidin (1.5% celloidin for 1 week, 3% for 2 weeks, 6% for 3 weeks, 12% for 3 weeks). Hardened ears were mounted on a fiber block for sectioning with a sliding microtome. The sections were mounted on glass slides, stained with H&E, and preserved in 80% alcohol. Imaging of these sectioned specimens was conducted with an Olympus BH2 microscope (Olympus, Tokyo, Japan) at 2× and 10× magnifications for Fig. 1a,b, respectively. Figure 1b was cropped using Adobe Photoshop CS5.1 (Adobe, Inc., San Jose, CA) to highlight cellular detail (Fig. 1c).

### Immunohistochemistry and confocal imaging

Guinea pig cochleae were extracted, fixed, and decalcified as described above. Under a stereomicroscope, the decalcified otic capsule was peeled away from the cochlea, and the 4 turns of the cochlea were sectioned into eight pieces. Each piece was further microdissected to reveal the organ of Corti. The spiral ligament, stria vascularis, and tectorial and Reissner’s membranes were removed.

After being rinsed in PBS for 15 minutes, cochlear sections were blocked with 5% Normal Horse Serum (NHS; Sigma-Aldrich, St. Louis, MO) in 1% Triton X-100 (Integra Chemical, Kent, WA), and were placed on a shaker for 30 minutes at room temperature. Sections were incubated with a primary antibody against neurofilament-H (polyclonal chicken; AB5539, Lot #2701573; EMD Millipore, Temecula, CA) overnight. After rinsing for 15 minutes in PBS, the tissue was incubated in a secondary antibody (AlexaFluor 488 goat anti-chicken IgG, A-11039, Lot #898239; ThermoFisher Scientific, Inc., Waltham, MA) diluted in 1% NHS with 0.4% Triton X-100 for 90 minutes in combination with 1:200 rhodamine phalloidin (ThermoFisher Scientific, Waltham, MA). Finally, the tissue was placed in Hoechst stain 33342 (Life Technologies, NY; 1 nM in PBS), and washed for 15 minutes in PBS and briefly in distilled water. Stained tissue was mounted under coverslips on glass slides with Vectashield mounting media (Vector Laboratories, CA, #H-1000).

Cochlear whole mounts were imaged with 20X (PlanApochromat, oil immersion, NA=0.7; #H1LG/02; Leica, Wetzlar, Germany) and 63X (PlanApochromat, oil immersion, NA=1.3, #506194; Leica, Wetzlar, Germany) Leica objectives, using a Leica TCS SP5 laser-scanning confocal microscope. Images (1024 x 1024 pixels) were collected in z-stacks and masked as maximum intensity projection images in the Leica software. The final images were cropped and scale bars were retraced in Photoshop CS5.1.

## Additional Information

**How to cite this article**: Iyer, J. S. *et al*. Micro-optical coherence tomography of the mammalian cochlea. *Sci. Rep.* **6**, 33288; doi: 10.1038/srep33288 (2016).

## Supplementary Material

Supplementary Video 1A

Supplementary Video 1B

Supplementary Video Legends
