# Supplementary material for: Micro-optical coherence tomography of the mammalian cochlea
Source: Sci Rep. 2016 Sep 16;6:33288. doi: 10.1038/srep33288 (PMC5025881; doi:10.1038/srep33288)
Supplement: Supplementary Video Legends [file srep33288-s3.doc]

**Title: Micro-optical coherence tomography of the mammalian cochlea**

**Authors: Janani S. Iyer, BA; Shelley A. Batts, PhD; Kengyeh K. Chu, PhD; Mehmet Ilhan Sahin, MD; Hui Min Leung, PhD; Guillermo J. Tearney, MD, PhD; Konstantina Stankovic, MD, PhD**

Supplemental Videos Legends

Visualization 1: Endoscopy perspective videos of reconstructed μOCT images of the space of Nuel (left; Visualization 1a) and tunnel of Corti (right; Visualization 1b), which reveal several bundles of spiral ganglion neurites traversing radially towards the outer hair cell region. Composed of 200 frames, captured at 15 fps; 500 μm x 500 μm field of view.
